# Digital health needs for long-term continuity care in older adult patients: a cross-sectional study under the “internet +” model

**DOI:** 10.3389/fpubh.2026.1763772

**Published:** 2026-05-04

**Authors:** ChunLing Sun, MinJie Ji, Fang Zhao, Xin Hu, BingQing Wang

**Affiliations:** Department of Neurology, Taiyuan Central Hospital, Taiyuan, Shanxi, China

**Keywords:** continuous nursing, cross-sectional study, digital health, older adult patients, Internet +, needs assessment

## Abstract

**Objective:**

The “Internet+” initiative offers new possibilities for addressing the complex health needs of aging populations. This cross-sectional study aims to investigate the demands for long-term continuous care among older adult patients with neurological disorders and to identify the key factors influencing these needs within the context of a digital health framework.

**Methods:**

In August 2024, a cross-sectional survey was conducted among hospitalized neurology patients from several Grade III-A hospitals in Taiyuan City, Shanxi Province. Using a convenience sampling method, data were collected through standardized questionnaires administered via the NetSun electronic platform. The survey captured patient demographics, disease characteristics, and their specific needs for out-of-hospital long-term care services. Univariate analysis and multivariable logistic regression were employed to identify factors associated with the demand for continuous care.

**Results:**

A total of 309 patients were surveyed, including 146 requiring long-term care outside hospitals and 163 not needing it. Among the required nursing services, medication management and lifestyle guidance ranked highest at 77.67% and 66.99%, respectively, while rehabilitation training accounted for 62.14%. Psychological intervention showed the lowest demand at 38.83%. Multivariable logistic regression analysis revealed that history of hospitalization (comparing patients with no history of hospitalization to those with a history, OR = 0.364, 95% CI: 0.142–0.937, *P* = 0.037) and degree of daily life impairment (with the odds ratio indicating a decreasing likelihood of needing care as impairment severity decreases, OR = 0.348, 95% CI: 0.249–0.487, *p* < 0.001) were significant predictors of the demand for continuous care, while smart device usage was not significant (OR = 1.231, 95% CI: 0.669–2.265, *P* = 0.500). The findings indicate that within the “Internet+” ecosystem, older adults demonstrate substantial needs for sustained long-term care models, particularly those with prior hospitalizations and greater functional impairment.

**Conclusion:**

Older adult patients with neurological disorders exhibit significant demand for post-discharge continuous care, particularly for medication management and lifestyle guidance. The need for care is strongly associated with clinical factors such as hospitalization history and functional impairment, rather than digital access alone. These findings underscore the importance of developing targeted, need-based digital health interventions to support this vulnerable population.

## 1 Introduction

The rapid advancement of internet technology has positioned the “Internet Plus” initiative as a transformative force across various industries, including healthcare (1–3). Its core concept involves the deep integration of internet technologies with traditional sectors to foster innovation, enhance efficiency, and improve service delivery (4, 5). In the context of an aging global population, this paradigm offers unprecedented opportunities to reshape healthcare services, particularly for the growing number of older adults requiring long-term support.

The world is facing an unprecedented demographic shift, with populations aging at an accelerating rate. In China, this trend is particularly pronounced; data from the Seventh National Population Census reveal that individuals aged 65 and above now constitute 13.5% of the national population, a figure that has risen by 4.63 percentage points since 2010 (6). This translates to hundreds of millions of older adults, many of whom live with chronic conditions, disabilities, or functional limitations that necessitate ongoing support (7, 8). For these individuals, the continuity and quality of care are not merely matters of convenience but are critical determinants of their health outcomes, functional independence, and overall quality of life (9). Consequently, developing sustainable and effective long-term care (LTC) models has become a paramount public health priority.

Traditional LTC, typically provided in institutional settings, is increasingly being supplemented by extended care models that bridge hospital treatment and home-based recovery. These models aim to provide post-discharge support, reduce hospital readmissions, and promote sustained patient engagement. However, the implementation of extended care remains fraught with challenges, including care coordination gaps, variable patient compliance, and limited professional oversight (10–12).

The “Internet Plus” initiative, which promotes the deep integration of internet technologies with traditional industries, offers a promising avenue to address these challenges. By leveraging digital platforms, smart devices, and real-time communication, it is possible to create a more seamless and integrated care continuum that connects hospitals, communities, and homes (1, 2, 4). This has the potential to enhance resource allocation, improve accessibility, and deliver personalized care at scale.

Despite the conceptual appeal of “Internet Plus” nursing, the evidence base required for its effective deployment is still nascent. A critical prerequisite for designing any service, digital or otherwise, is a thorough understanding of the target population's needs. Several recent studies have explored “Internet Plus” nursing models (3, 5, 13), but they have often focused on pilot interventions for specific diseases or have been largely descriptive. What is lacking is robust, quantitative evidence that systematically analyzes the specific LTC needs of older adult patients and, more importantly, identifies the key determinants of those needs from a demand-side perspective. From a health economics and policy standpoint, the relevant gap is not the absence of a scalable digital model *per se*, but rather the lack of empirical data quantifying the factors-such as clinical status, functional ability, and socioeconomic conditions-that drive the demand for different types of care. Without this foundational knowledge, efforts to build digital systems risk being misdirected or inefficient.

Therefore, this study aims to address this gap by conducting a quantitative cross-sectional analysis of LTC needs among a cohort of older adult patients with neurological disorders. Our primary objectives are: (1) to describe the demographic and clinical profile of these patients and quantify their demand for various out-of-hospital continuous care services; and (2) to identify the key predictors of this demand using multivariable regression analysis. By providing empirical evidence on what older adult patients need and what factors influence those needs, this study offers a crucial foundation for the development of targeted, evidence-based, and patient-centered digital health strategies. This contribution moves the discourse from broad technological possibilities to specific, actionable insights for policy and practice, ultimately aiming to inform the design of more effective and sustainable LTC systems within the “Internet Plus” framework.

## 2 Objects and methods

### 2.1 Survey subjects

A cross-sectional study was conducted in August 2024. Using a convenience sampling method, we recruited inpatients from the neurology departments of several Grade III-A hospitals in Taiyuan City, Shanxi Province.

Inclusion criteria:

① Age ≥ 60 years;
② Diagnosed with one of the following neurological diseases: cerebral infarction, cerebral hemorrhage, epilepsy, Parkinson's disease, or dementia;
③ Both patients and their family members (or legally authorized representatives in cases of limited patient capacity) provided informed consent and voluntarily participated in the study.

Exclusion criteria:

① Patients with severe complications affecting the heart, lungs, liver, or kidneys;
② Those with neurological diseases not related to the study focus (e.g., peripheral neuropathy, myasthenia gravis, multiple sclerosis);
③ Patients with diagnosed mental disorders or cognitive impairment severe enough to preclude meaningful participation.

This study has been approved by the ethics committee of Taiyuan Hospital, Peking University First Hospital.

### 2.2 Survey tools and measures

A structured questionnaire was developed by the research team based on a review of the literature on long-term care needs and “Internet Plus” nursing services. The questionnaire consisted of four sections: Socio-demographic characteristics: This section collected data on age, gender, and educational level. Clinical characteristics: Patients reported their primary disease diagnosis (cerebral infarction, cerebral hemorrhage, epilepsy, Parkinson's disease, or dementia), history of hospitalization (yes/no), and the degree to which their disease impacts daily life. The degree of life impairment was assessed using a single-item, self-report question with four ordinal response categories: “severe,” “moderate,” “mild,” or “none.” Digital literacy: Patients were asked about their use of smart devices (yes/no), defined as owning and being able to independently operate a smartphone or tablet. Need for out-of-hospital long-term care: The core dependent variable was assessed by asking patients: “Do you need long-term care outside the hospital?” (yes/no). For those responding “yes,” they were further asked to select their specific needs from a pre-defined list of services, including medication management, lifestyle guidance, rehabilitation training, and psychological intervention. Multiple selections were permitted. The questionnaire was piloted with a small sample of 20 patients (not included in the main study) to check for clarity and comprehension, leading to minor wording adjustments. However, formal tests for reliability and validity, such as test-retest reliability or confirmatory factor analysis, were not conducted for this survey, which is a limitation of this study.

### 2.3 Data collection and quality control methods

The research team underwent standardized training prior to conducting the survey. After coordinating with head nurses of neurology departments at various hospitals, they distributed questionnaires through the NetSun electronic platform to all clinical departments. Before data collection, a unified script was used to explain the study's objectives, significance, and precautions, with real-time online consultations provided for nurses' inquiries. Participants completed the questionnaires anonymously on the platform. A feature of the electronic platform required participants to answer all questions before submission, which minimized item non-response. Post-collection, the data was exported and cross-checked by a dual-team verification process. Any discrepancies or questionable entries were promptly addressed through follow-up communication with participants to ensure data accuracy.

### 2.4 Statistical methods

Data were entered into Excel and analyzed using SPSS version 26.0 (IBM Corp., Armonk, NY, USA). Categorical variables were summarized as frequencies and percentages. Continuous variables were described using means and standard deviations. To examine factors associated with the need for out-of-hospital long-term care, we first conducted univariate analyses using the Chi-square test for categorical independent variables (e.g., age group, gender, history of hospitalization) against the binary dependent variable (need for care: yes/no). Variables with a *P*-value < 0.10 in the univariate analysis, along with key demographic variables (age, gender) considered theoretically important, were entered into a multivariable binary logistic regression model. This model is appropriate for a binary outcome. The results are presented as odds ratios (ORs) with 95% confidence intervals (CIs) and *P*-values. A *P*-value of less than 0.05 was considered statistically significant.

## 3 Results

### 3.1 General characteristics of the study participants

A total of 309 questionnaires were distributed and collected. Due to the forced-response feature of the electronic data collection platform, there were no missing data or logical errors, resulting in a 100% valid response rate for the distributed questionnaires. However, it is important to acknowledge that this high rate may also reflect a selection bias inherent in the convenience sampling method. The general characteristics of the participants are presented in Table 1. The majority of participants were aged 60–65 years (36.89%), followed by those aged 71–75 years (23.95%). Gender distribution was relatively balanced, with slightly more females (52.10%) than males (47.90%). Most participants had a relatively low educational level, with 24.92% having primary school education or below and 35.28% having junior middle school education.

**Table 1:** Demographic characteristics of the study participants (*N* = 309).

Variable	Category	Frequency	Percentage (%)
Age (years)	60–65	114	36.89
	66–70	62	20.06
	71–75	74	23.95
	≥76	59	19.09
Gender	Male	148	47.90
	Female	161	52.10
Education level	Primary or below	77	24.92
	Junior middle school	109	35.28
	Senior middle school	49	15.86
	Special school	24	7.77
	College or above	50	16.18

### 3.2 Disease distribution and long-term care needs

**Figure 1:**
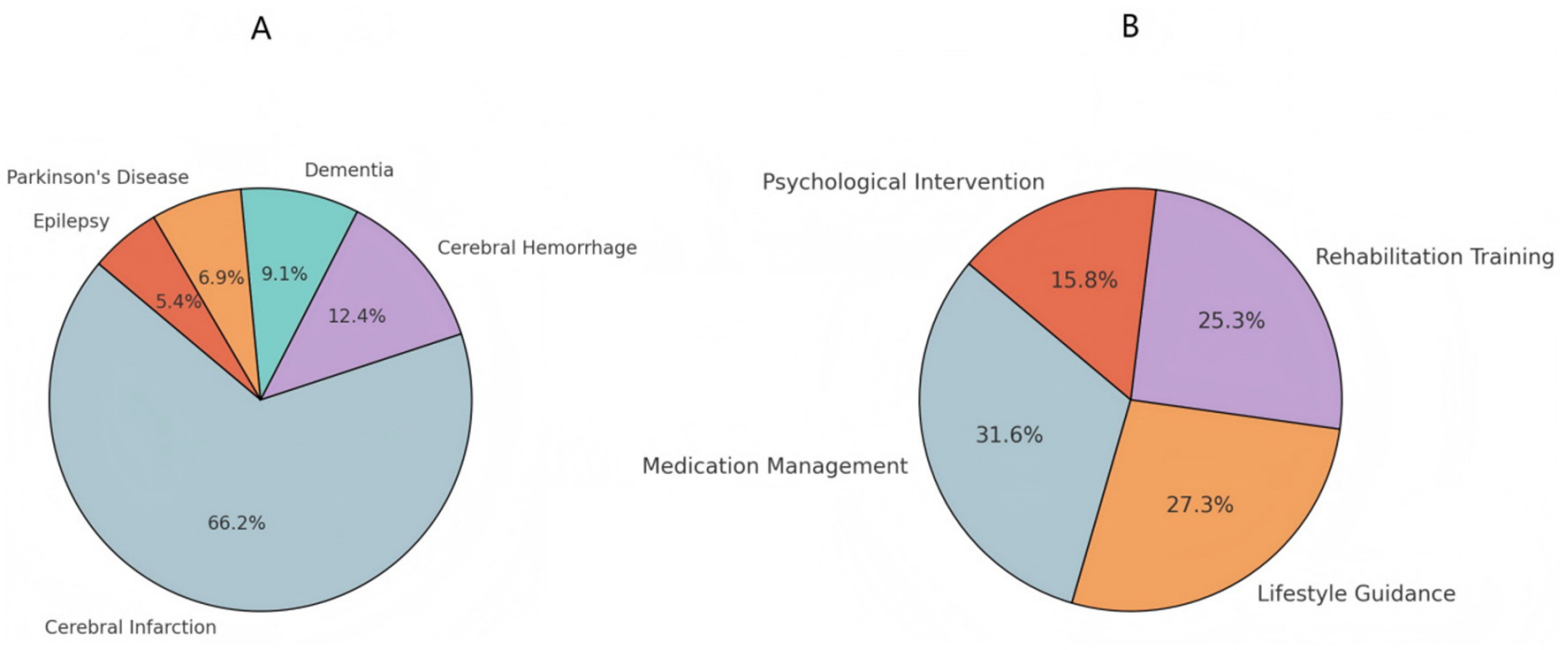
Disease distribution and long-term care needs. **(A)** What is the name of your disease diagnosis? **(B)** Which aspects of long-term care do you need.

As shown in Figure 1A, cerebral infarction was the most common diagnosis (70.87%), followed by cerebral hemorrhage (13.27%), dementia (9.71%), Parkinson's disease (7.44%), and epilepsy (5.83%). Analysis of long-term care needs (Figure 1B) indicated that medication management was the most required service (77.67%), followed by lifestyle guidance (66.99%) and rehabilitation training (62.14%). Psychological intervention had the lowest demand (38.83%).

### 3.3 Univariate analysis of factors associated with out-of-hospital long-term care needs

Univariate analysis using the Chi-square test revealed significant associations between the need for long-term out-of-hospital care and the following factors: history of hospitalization (χ^2^ = 11.759, *P* = 0.001), degree of daily life impairment (χ^2^ = 65.141, *P* < 0.001), and use of smart devices (χ^2^ = 4.627, *P* = 0.031). No significant differences were observed regarding age, gender, or educational background (all *P* > 0.05). Detailed results are presented in Table 2.

**Table 2:** Univariate analysis of factors associated with the need for long-term out-of-hospital care.

Variable	Category	Do you need long-term care outside the hospital? n (%)		Total	χ2	*p*
		Yes	No			
Age (years)	60–65	49 (42.98)	65 (57.02%)	114	1.613	0.656
	66–70	32 (51.61)	30 (48.39%)	62		
	71–75	35 (47.30)	39 (52.70%)	74		
	≥76	30 (50.85)	29 (49.15%)	59		
Gender	Male	74 (50.00)	74 (50.00%)	148	0.862	0.353
	Female	72 (44.72)	89 (55.28%)	161		
Education level	Primary or below	46 (59.74)	31 (40.26%)	77	8.279	0.082
	Junior middle school	46 (42.20)	63 (57.80%)	109		
	Senior middle school	18 (36.73)	31 (63.27%)	49		
	Special school	11 (45.83)	13 (54.17%)	24		
	College or above	25 (50.00)	25 (50.00%)	50		
History of hospitalization	Yes	139 (50.73)	135 (49.27%)	274	11.759	0.001^**^
	No	7 (20.00)	28 (80.00%)	35		
Degree of life impairment	Severe	42 (89.36)	5 (10.64%)	47	65.141	< 0.001^**^
	Moderate	52 (59.09)	36 (40.91%)	88		
	Mild	41 (36.61)	71 (63.39%)	112		
	None	11 (17.74)	51 (82.26%)	62		
Use of smart devices	Yes	104 (43.88)	133 (56.12%)	237	4.627	0.031^*^
	No	42 (58.33)	30 (41.67%)	72		

^*^p < 0.05, ^**^p < 0.01.

### 3.4 Multivariable logistic regression analysis of factors associated with care needs

The results of the multivariable binary logistic regression analysis are presented in Table 3. After adjusting for other variables in the model, two factors emerged as significant independent predictors of the need for out-of-hospital long-term care. Patients with no history of hospitalization had significantly lower odds of needing care compared to those with a hospitalization history (OR = 0.364, 95% CI: 0.142–0.937, *P* = 0.037). Furthermore, the degree of daily life impairment was a strong and significant predictor. In our model, where the impairment scale was coded ordinally with higher values indicating less severe impairment (1 = severe, 2 = moderate, 3 = mild, 4 = none), the odds ratio was 0.348 (95% CI: 0.249–0.487, *P* < 0.001). This means that for each one-level decrease in impairment severity (e.g., from ‘severe' to ‘moderate', ‘moderate' to ‘mild', or ‘mild' to ‘none'), the odds of needing care were reduced by approximately 65%. Conversely, this finding robustly indicates that patients with more severe functional impairment were much more likely to require out-of-hospital long-term care. The use of smart devices was not a statistically significant predictor in the multivariable model (OR = 1.231, 95% CI: 0.669–2.265, *P* = 0.500) (18).

**Table 3:** Multivariable binary logistic regression analysis of factors associated with the need for long-term out-of-hospital care (*N* = 309).

Variable	Category	β	SE	Wald χ^2^	*P*-value	Odds ratio (OR)	95% Confidence interval for OR
History of hospitalization	Yes (ref)					1.00	
	No	−1.011	0.484	4.366	0.037	0.364	0.142-−0.937
Degree of life impairment	(Ordinal scale: none/mild/moderate/severe)	−1.054	0.154	46.796	< 0.001	0.348	0.249-−0.487
Use of smart devices	Yes (ref)					1.00	
	No	0.208	0.308	0.455	0.500	1.231	0.669-−2.265
Constant		3.476	0.783	19.719	< 0.001	32.334	Constant
Model Fit Indices	Value						
Hosmer-Lemeshow Test	χ^2^ = 4.867, df = 8, *P* = 0.772						
Nagelkerke *R*^2^	0.357						
−2 Log Likelihood	312.845						

Dependent variable: need for long-term out-of-hospital care (yes/no). The analysis was adjusted for age and gender, which were not significant predictors. ref = reference category. The Hosmer-Lemeshow test indicates good model fit (*p* > 0.05). The odds ratio for degree of life impairment represents the change in odds for each one-level increase in impairment severity (from none to mild, mild to moderate, or moderate to severe). A lower odds ratio indicates higher likelihood of needing care.

## 4 Discussion

This cross-sectional study investigated the digital health needs for long-term continuous care among 309 older adult patients with neurological disorders in Taiyuan, China. Our findings reveal a substantial demand for out-of-hospital services, with medication management (77.67%) and lifestyle guidance (66.99%) being the most frequently cited needs. More importantly, the multivariable analysis identified history of hospitalization and the degree of daily life impairment as the strongest independent predictors of care demand, while smart device usage was not a significant factor. These results have important implications for the design and targeting of “Internet Plus” nursing services.

The high demand for medication management and lifestyle guidance aligns with the chronic nature of the conditions in our sample, predominantly cerebrovascular diseases. For patients with cerebral infarction or hemorrhage, adherence to complex medication regimens and secondary prevention through lifestyle modification are critical for preventing recurrence and managing disability. The lower demand for psychological intervention (38.83%) is an intriguing finding that warrants deeper exploration. While it could suggest a lower perceived need for mental health support, several alternative explanations are plausible within the Chinese cultural context. First, there may be a significant cultural stigma associated with mental health issues, leading patients to underreport psychological distress or to somaticize their symptoms, expressing them as physical complaints rather than emotional ones. Second, there might be a lack of health literacy regarding psychological wellbeing; patients and their families may not recognize symptoms of depression or anxiety as treatable conditions, or they may prioritize physical rehabilitation over mental health. Third, it is possible that the single-item measure (“need for psychological intervention”) was too blunt to capture the nuanced nature of psychosocial support needs. Future research should employ validated mental health screening tools to objectively assess the prevalence of depression and anxiety in this population, alongside qualitative interviews to understand patients' perceptions and help-seeking behaviors regarding psychological care. The low reported demand does not necessarily indicate an absence of need, but rather a potential gap in awareness or a barrier to disclosure.

The regression analysis provides critical insights for designing targeted digital health interventions. The strong association between care need and both hospitalization history and functional impairment underscores that the demand for post-discharge support is fundamentally driven by clinical and functional status. Patients with recent hospitalizations and those reporting greater difficulty in daily life are the ones who most acutely feel the need for ongoing professional support. This suggests that “Internet Plus” nursing services should be strategically targeted at these high-risk groups upon discharge, rather than being offered as a universal, one-size-fits-all solution.

Importantly, the use of smart devices was not a significant predictor of care need in the multivariable model. While digital literacy is an enabler for accessing online services, it does not, by itself, create demand for care. This finding challenges a simplistic, technology-driven view of digital health and reinforces the principle that need, rather than technological access, should be the primary driver of service design. Many older adult non-users of smart devices still expressed a high need for care, highlighting the danger of a “digital divide” where those who need services most could be excluded if interventions rely solely on app-based platforms. Therefore, a successful “Internet Plus” model must be hybrid and inclusive. It should integrate digital tools (e.g., apps for medication reminders, video consultations for those who can use them) with traditional, low-tech methods of communication and support, such as telephone follow-ups, home visits by community nurses, and involvement of family caregivers. The platform should serve as a coordination hub, but the delivery of care must be flexible enough to accommodate varying levels of digital literacy.

Our findings both align with and extend previous research. The high demand for practical, daily management support (medication, lifestyle) is consistent with studies on chronic disease management in aging populations (16, 17). However, our study adds to the literature by quantitatively demonstrating the primacy of clinical factors over demographic or technological ones as determinants of care need. While other studies have explored patient satisfaction with “Internet Plus” pilots (11, 12, 14), our research provides foundational evidence on the demand-side characteristics that should inform the targeting and content of such programs.

This study has several limitations that should be considered when interpreting the findings. First, its cross-sectional design precludes any causal inferences; the associations identified between predictors and care needs are correlational only. Second, the use of convenience sampling from hospitals in a single city (Taiyuan) may limit the generalizability of our findings to the broader population of older adult patients in China (9). Third, the data on care needs, clinical characteristics, and digital literacy were all based on self-report, which is subject to recall bias and social desirability bias (13). Fourth, and importantly, the questionnaire used was self-developed and did not undergo formal validation (e.g., test-retest reliability, construct validity). While it was based on a literature review and piloted for clarity, the lack of psychometric testing means that the precision and accuracy of our measurements are unknown, and this represents a significant methodological weakness (15). Fifth, our regression model suffered from potential omitted variable bias. Key factors theorized to influence care demand, such as household income, health insurance type, living arrangements (e.g., living alone vs. with family), and more nuanced measures of digital health literacy, were not collected (17). Their omission could mean that the estimated effects of the included variables are biased. Finally, the study focused on patients with specific neurological disorders; the findings may not be applicable to older adult patients with other types of chronic conditions (7, 8).

## 5 Conclusion

This cross-sectional study provides empirical evidence on the digital health needs for long-term continuous care among older adult patients with neurological disorders. Our findings reveal a substantial demand for out-of-hospital services, particularly for medication management and lifestyle guidance. Importantly, the need for care was most strongly associated with clinical factors-specifically, a history of hospitalization and the degree of daily life impairment-rather than with demographic characteristics or smart device usage alone. This suggests that while digital platforms are essential tools for service delivery, the foundation of care need is rooted in patients' clinical and functional status. These results highlight the importance of developing targeted, need-based digital health interventions. Future prospective, longitudinal research is warranted to track how these needs evolve over time and to evaluate the effectiveness and cost-effectiveness of specific “Internet Plus” care models in improving patient outcomes and quality of life. From a policy perspective, efforts should focus on integrating digital platforms with community-based nursing resources to create a flexible, inclusive care continuum that can adapt to the diverse clinical and digital literacy profiles of the aging population. Rather than assuming positive attitudes toward technology, interventions should be designed based on documented needs, ensuring that services reach those with the greatest clinical requirements, regardless of their digital proficiency.

## Data Availability

The original contributions presented in the study are included in the article/supplementary material, further inquiries can be directed to the corresponding author.
